# Decreased Fronto-Temporal Interaction during Fixation after Memory Retrieval

**DOI:** 10.1371/journal.pone.0110798

**Published:** 2014-10-23

**Authors:** Masaki Katsura, Satoshi Hirose, Hiroki Sasaki, Harushi Mori, Akira Kunimatsu, Kuni Ohtomo, Koji Jimura, Seiki Konishi

**Affiliations:** 1 Department of Radiology, The University of Tokyo School of Medicine, Tokyo, Japan; 2 Department of Physiology, The University of Tokyo School of Medicine, Tokyo, Japan; 3 Department of Physiology, Juntendo University School of Medicine, Tokyo, Japan; 4 Precision and Intelligence Laboratory, Tokyo Institute of Technology, Yokohama, Japan; University of Michigan, United States of America

## Abstract

Previous studies have revealed top-down control during memory retrieval from the prefrontal cortex to the temporal cortex. In the present functional MRI study, we investigated whether the fronto-temporal functional interaction occurs even during fixation periods after memory retrieval trials. During recency judgments, subjects judged the temporal order of two items in a study list. The task used in the present study consisted of memory trials of recency judgments and non-memory trials of counting dots, and post-trial fixation periods. By comparing the brain activity during the fixation periods after the memory trials with that during the fixation periods after the non-memory trials, we detected heightened brain activity in the lateral prefrontal cortex, the lateral temporal cortex and the hippocampus. Functional interactions during the fixation periods after the memory vs. non-memory trials as examined using a psychophysiological interaction revealed a decreased interaction from the lateral prefrontal cortex to the lateral temporal cortex, but not to the hippocampus. The functional interaction between the same frontal and temporal regions was also present during the memory trials. A trial-based functional connectivity analysis further revealed that the fronto-temporal interaction was positive and decreased during the fixation periods after the memory trials, relative to the fixation periods after the non-memory trials. These results suggest that the fronto-temporal interaction existed during the post-trial fixation periods, which had been present during the memory trials and temporally extended into the fixation periods.

## Introduction

The lateral prefrontal cortex has been implicated in various types of cognitive control that guides our behavior, including memory control. The contribution of the lateral prefrontal cortex to control of memory retrieval has widely been acknowledged, such as selection or suppression of memory [1]–[17]. Previous studies also revealed that the memory representations in the temporal cortex were activated at the same time with the prefrontal cortex during memory retrieval [18]–[19], and that fiber density between prefrontal and temporal cortex predicted episodic memory performance [20], suggesting a top-down interaction from the prefrontal cortex to the temporal cortex.

Memory retrieval tasks are often designed as the alternation of memory trials and inter-trial intervals of post-trial fixation periods used as a control for memory retrieval. It is possible that memory retrieval processes and fronto-temporal interaction continue even after memory retrieval trials are completed, which may raise the possibility that the post-trial fixation periods may not be regarded as an ideal low-level control. In the present functional MRI study, we investigated whether functional interaction between the lateral prefrontal cortex and the temporal cortex exists even after memory retrieval trials. A recency judgment task was employed where two studied items were judged as to which was presented more recently [21]–[44]. The task consisted of recency judgment trials, non-memory trials of counting dots, and post-trial fixation periods of the same durations (3 sec each). The recency judgment task can be expected to require retrieval of greater amount of episodes for judgment of temporal order of studied items, which might resulted in greater degree of recruitment of processing related to memory retrieval even after the memory trials are completed. By comparing the brain activity during the post-trial fixation periods that followed the recency judgment trials with that during the post-trial fixation periods that followed the non-memory trials, we detected heightened brain activity. Further, a functional interaction among the brain activations identified in the prefrontal and temporal cortex was examined using a psychophysiological interaction (PPI) [45]–[46] analysis. We also used resting-state data to contrast with the post-trial fixation periods where control processing was enhanced.

## Materials and Methods

### Subjects

Written informed consent was obtained from 31 healthy right-handed subjects (18 males; 13 females, age: 20–29 years). Three experiments were performed in a 2-hour session (Exp. 1: recency judgments; Exp. 2: dot counting and control fixation, Exp. 3: resting state). In the third experiment, 5 subjects did not complete the experiment due to the limitation of scanner time, and data from the 26 subjects were analyzed. They were scanned using experimental procedures approved by the institutional review board of the University of Tokyo School of Medicine.

### MRI procedures

The experiments were conducted using a 3T fMRI system. Scout images were first collected to align the field of view centered on the subject’s brain. T1-weighted images were obtained for anatomical reference (76 slices×2 mm slices; in-plane resolution = 1×1 mm). For functional imaging, a gradient echo echo-planar sequence was used (TR = 2.0 s; TE = 35 ms; flip angle = 90 degrees; 30×4 mm slices; in-plane resolution of 4×4 mm). Each run contained 36 volume images, and the first six functional images in each run were excluded from the analysis to take into account the equilibrium of longitudinal magnetization.

### Behavioral Procedures

**Figure 1:**
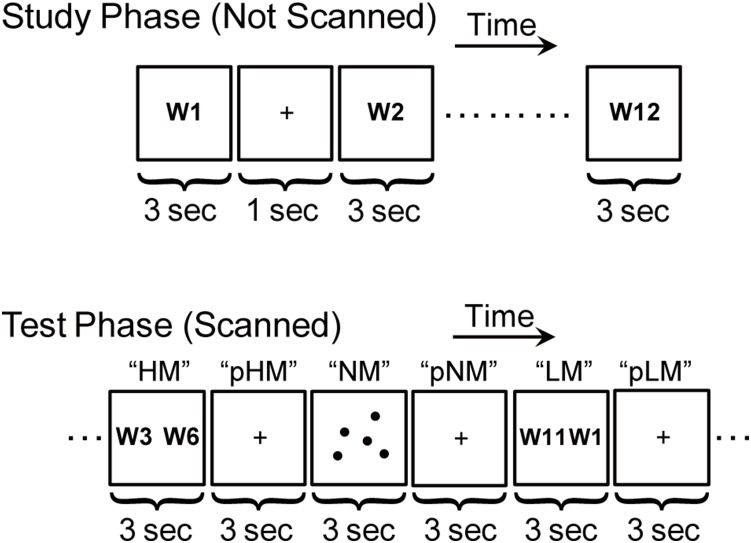
The memory paradigm of the present study. The task in the test phase contained memory trials of recency judgments with high and low retrieval loads (HM and LM), non-memory trials of counting dots (NM), and post-trial fixation periods (pHM, pLM and pNM) that lasted 3 sec each. W: word.

The task in Exp. 1 consisted of two main phases, study and test (Fig. 1). During the study phase, the subjects were presented with a sequence of words (list size: 12 words). Each word was presented for 3 sec, with an inter-stimulus interval (presentation of a white fixation cross) of 1 sec. Subjects were instructed to relationally encode them for later recency judgments [33], [47]–[48]. More specifically, subjects were instructed to make up their own story from the list words, and this instruction is supposed to encourage the subjects to relate sequentially presented words that had otherwise no contexts among them. The words were concrete nouns taken from an object stimulus set [49], and were presented in strings of Japanese characters. To prevent the subjects from rehearsing the words between the study and test phases, the subjects performed a modified Wisconsin card sorting task for approximately 30 s as a distracter task [28], [33], [41], [50].

The test phase was administered while functional images were acquired. In each of twelve runs administered to the subjects, the test phase contained four recency judgment trials with high or low mnemonic load (two “HM” and two “LM” trials) and four non-mnemonic odd/even judgment trials (“NM”), presented in a pseudorandom order. Therefore, the total numbers of HM, LM and NM trials were 24, 24 and 48, respectively. Each trial lasted for 3 sec, followed by a 3-sec fixation period, which consisted of three types of post-trial fixation periods (“pHM”, “pLM” and “pNM”). During the recency judgment trials (HM and LM), two words in the studied list were simultaneously presented, one to the right and the other to the left. The subjects were instructed to choose which word had been studied more recently. The right or left word was chosen by pressing a right or left button, respectively, using the same right thumb. The word pair to be judged for LM trials included one or two end words in the study list, and the temporal distance between the paired words was greater, whereas the word pair for HM trials did not include any end words, and the temporal distance between the paired words was smaller. By contrast, during the odd/even judgment trials (NM), subjects were instructed to count 3 to 8 white dots presented in the screen and to judge whether the number of the dots was even or odd. The even or odd number was chosen by pressing a right or left button, respectively, using the same right thumb. Therefore, HM and LM trials required memory retrieval processes to a greater degree than NM trials. During the post-trial fixation period, on the other hand, subjects were instructed to fixate on a cross presented at the center of the screen, but were not encouraged or discouraged to think about a particular thing. In order to avoid unwanted confound related to the difference in task performance prior to the fixation periods, the accuracy and reaction time in LM and NM trials were matched by modulating the number of dots in NM trials. Therefore, the contrast of pLM vs. pNM of central interest in the present study is expected to reveal the brain activity associated with memory-related processes after the memory trials. In other words, we focused on the pLM vs. pNM effect because, unlike the high memory conditions, this comparison was not confounded by reaction time or difficulty difference.

It is possible that a relatively weaker level of memory-related processes was recruited during the pNM presented in the runs that included recency judgment trials (HM and LM). In order to control for the possible confound, two runs of a control task were administered where five NM trials were presented in a block for 16 sec, followed by a fixation period for 16 sec, and the cycles were repeated five times for each run (Exp. 2). In addition, in order to examine the resting-state functional connectivity between the frontal and temporal regions of interest (ROIs), four runs were administered where subjects fixated on a cross hair throughout the run, approximately for 5 min (Exp. 3). The resting-state runs were not completed in 5 of the 31 subjects due to limitation in scanner time, and data from the 26 subjects were analyzed for the resting-state functional connectivity.

### Data analysis

Data were analyzed using SPM8 software (http://www.fil.ion.ucl.ac.uk/spm/). Functional images were realigned, slice timing corrected, normalized to the Montreal Neurological Institute template with interpolation to a 2×2×2 mm space, and spatially smoothed (full width half maximum = 8 mm). Then event timing was coded into a general linear model (GLM) [51]. The eight types of events, correct trials (HM, LM and NM), error trials, and subsequent fixation periods (pHM, pLM, pNM and post-error periods), together with run-specific regressors as effects of no interest, were coded using the canonical hemodynamic response function in SPM8, time-locked to the onset of these events. The brain activation associated with memory-related processes during the post-trial fixation periods was calculated based on the contrast of pLM vs. pNM. Group analyses were conducted using a random effects model. Significant activations were detected using a combined threshold of (1) p<0.05 corrected by the false discovery rate (FDR) [52], [53] and (2) p<0.001 (uncorrected).

A psychophysiological interaction (PPI) [45], [46] analysis was conducted to investigate functional interaction among brain regions during post-trial fixation periods. Based on the spherical ROIs (radius = 8 mm) determined by the post-trial contrast of pLM vs. pNM, PPIs from each of the ROIs in the left lateral prefrontal cortex to one ROI in the left temporal cortex and one ROI in the left hippocampus were calculated between two psychological conditions, pLM and pNM, in a single-subject level. A significant PPI means the regression of temporal responses depending on prefrontal top-down control processing in the low versus no-memory conditions. This regression is a simple linear form of effective connectivity and is interpreted as prefrontal influence on temporal processing. Group analyses were then conducted using a random effects model. In order to test the across-data reproducibility of the functional interaction during post-trial fixation periods, PPIs were also calculated between pHM and pNM based on the ROIs determined by the contrast of pLM vs. pNM, where PPIs had already been calculated between pLM and pNM.

Another type of a functional connectivity analysis was also performed on a trial by trial basis [54]. Briefly, each of the trials (HM/LM/NM) and post fixation periods (pHM/pLM/pNM) was coded into GLM, and the parameter estimate (beta value) for each trial in these six conditions was extracted. The beta values in one condition were plotted against two ROIs, and the correlation coefficient (r) across trials was calculated. The beta values in the two ROI were averaged across all the voxels in the sphere that were selected from the frontal and temporal regions activated during pLM minus pNM. The correlation coefficient (r) was converted to Fisher’s z values and then to z-score in a Gaussian distribution, and the z-score was subject to a t-test in a group analysis, to estimate a functional interaction between two ROIs in one condition.

The data analysis procedures for resting-state functional connectivity were essentially the same as those used in previous literatures [55]–[57]. Briefly, the acquired images were realigned, slice-timing corrected, and normalized to the standard template image. The images were subject to further preprocessing including temporal band-pass filter (0.009 Hz, f, 0.08 Hz), spatially smoothed, regression of six parameters obtained by head motion correction, whole brain signal averaged over the whole brain, ventricular signal averaged from ventricular ROI, and white matter signal averaged from white matter ROI. Functional connectivity analyses were performed on the resultant time series data, on a timepoint by timepoint basis, between a seed ROI and a target ROI or between a seed ROI and all the voxels in the whole brain. To estimate the statistical significance of the functional connectivity, the Fischer z transformation was applied to the correlation coefficients.

### Interview on the contents of thought during post-trial fixation period

In order to ensure that memory-related processes were recruited during the post-trial fixation periods, questionnaire about the content of thought during the fixation period was administered to a different set of 11 subjects (6 males, 5 females, age: 20–29 years). The subjects performed 5 runs of 2 LM and 2 NM trials (not scanned). After the task, the subjects were asked to report the contents of thought during fixation periods that followed LM or NM trials. The task was basically the same except that the task contained only LM and NM trials. HM trials were not included in this task because subjects cannot completely discriminate HM from LM at the time of questionnaire. More specifically, in the surprise report, they were asked to classify the contents of thought into four categories: (1) reflection on previous memory trials, (2) reflection on previous counting trials, (3) others and (4) no thinking activity. The scores were provided for pLM and pNM separately. The score ranged from 0 to 10 based on the time spent in that thought, and the sum of the four scores had to be ten.

## Results

### Behavioral results

**Figure 2:**
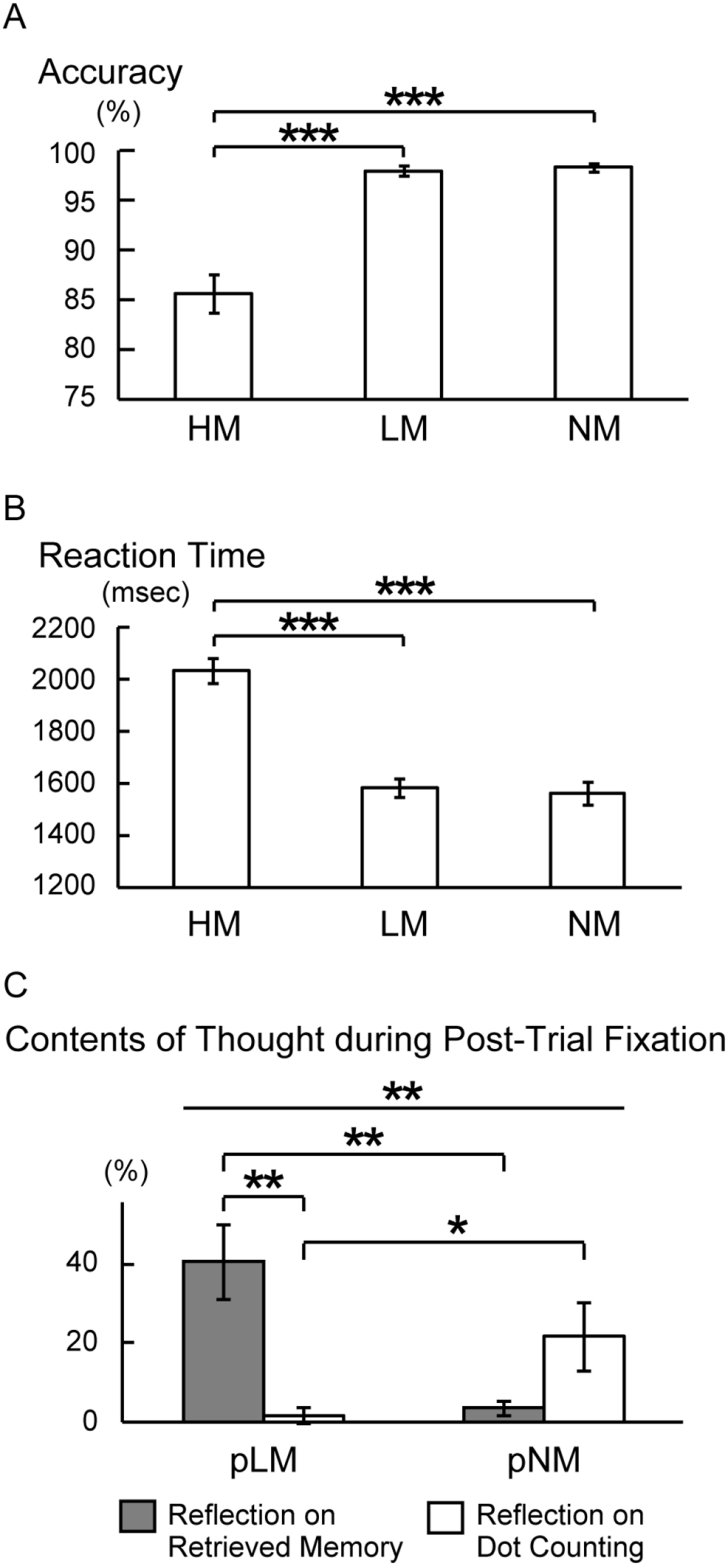
Behavioral data. A) Accuracy in the three types of trials, HM, LM and NM. B) Reaction time in correct trials in HM, LM and NM. C) Contents of thought during fixation periods after correct LM and NM trials. *: p<.05, **: p<.01, ***: p<.001.

The correct performance was 85.6±10.9 (mean ± SD), 98.0±2.8 and 98.3±2.4% in HM, LM and NM trials, respectively (Fig. 2A). The difference was significant between HM and LM trials [t (30) = 6.4, P<.001] and between HM and NM trials [t (30) = 6.4, P<.001]. The reaction time was 2034±265 (mean ± SD), 1582±198 and 1563±247 ms, in HM, LM and NM trials, respectively (Fig. 2B). The difference was significant between HM and LM trials [t (30) = 14.2, P<.001] and between HM and NM trials [t (30) = 10.6, P<.001]. However, no significant difference in the behavioral performance was found between LM and NM. Therefore, the behavioral scores were successfully matched between LM and NM.

Post-task questionnaire administered on a different set of subjects revealed that memory-related processes were recruited more during pLM than during pNM. Subjects reflected on previous memory trials during pLM for 41%, and reflected on previous counting trials during pNM for 22% (Fig. 2C). A repeated measures two-way ANOVA revealed significant interaction between trial-type and thought-content [F (1, 10) = 14.1, P<.005]. These results confirm that memory-related processes during memory trials were prolonged and were recruited during pLM.

### Neuroimaging results

**Figure 3:**
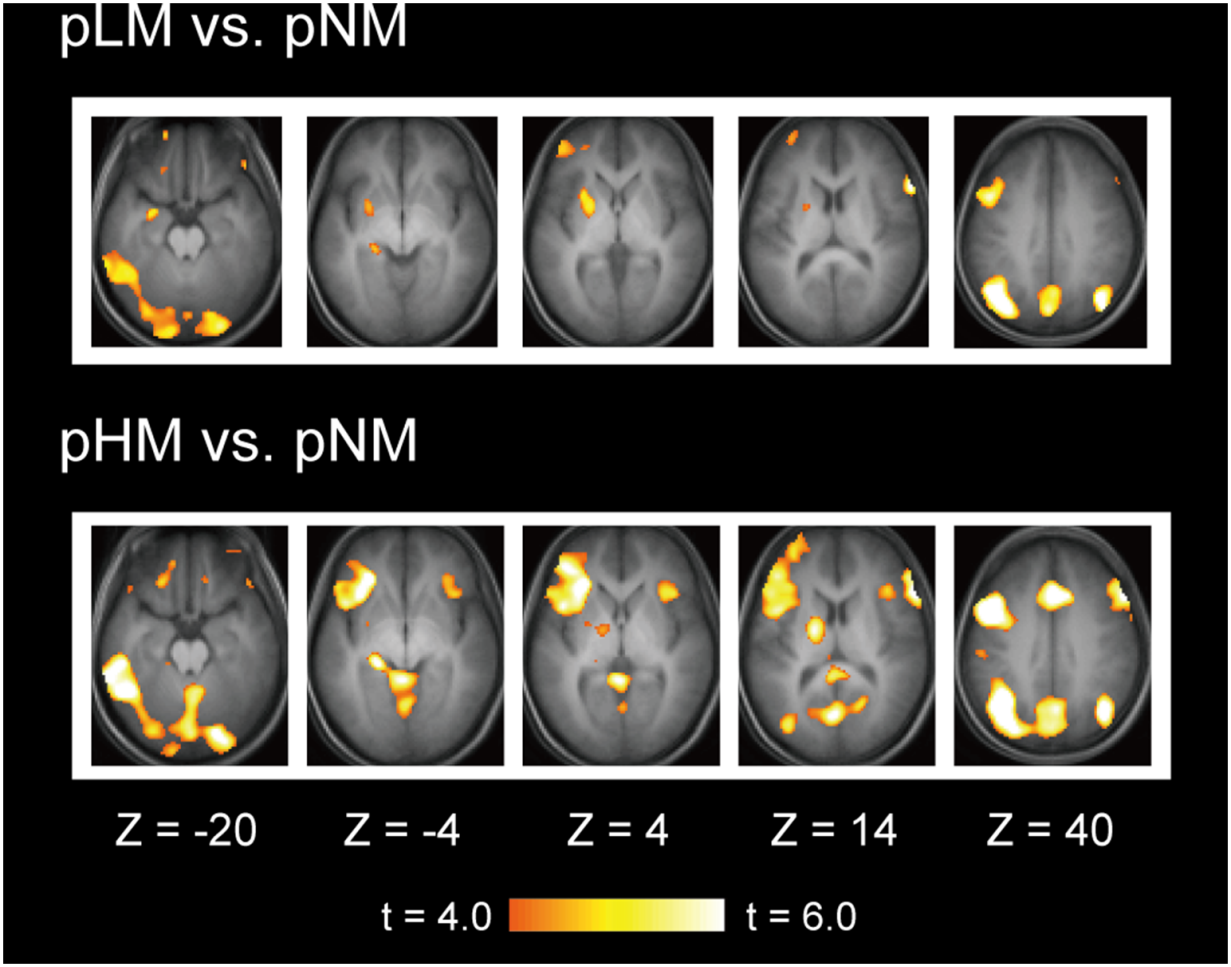
Statistical activation maps for signal increase in the contrast of pLM vs. pNM and pHM vs. pNM. Activation maps are displayed as transverse sections and are overlaid on top of the anatomic image averaged across subjects. Statistical significance is indicated using the color scale, and the transverse section level is indicated by the Z coordinates of Talairach space.

The brain activation associated with memory-related processes during the post-trial fixation periods was calculated based on the contrast of pLM/pHM vs. pNM. As shown in Fig. 3, significant signal increase was observed in several regions, including the regions in the left lateral prefrontal cortex, the left lateral temporal cortex and the left hippocampus. Table 1 lists the peak coordinates of the significant activations during pLM vs. pNM in the lateral prefrontal cortex and the temporal lobe that were used as regions of interest in later analyses.

**Table 1:** Brain regions in the left lateral prefrontal cortex and left temporal cortex showing signal increase in the contrasts pLM vs. pNM.

	X	y	z	t	Label
Left lateral prefrontal cortex	−50	10	40	5.8	Frontal1
	−42	22	44	5.1	Frontal2
	−46	50	4	4.7	Frontal3
	−34	58	14	4.5	Frontal4
	−28	50	4	4.2	Frontal5
	−46	46	18	4.2	Frontal6
Left temporal cortex	−66	−44	−20	5.0	Lat Temporal
	−22	−32	−4	4.7	Hippocampus

**Figure 4:**
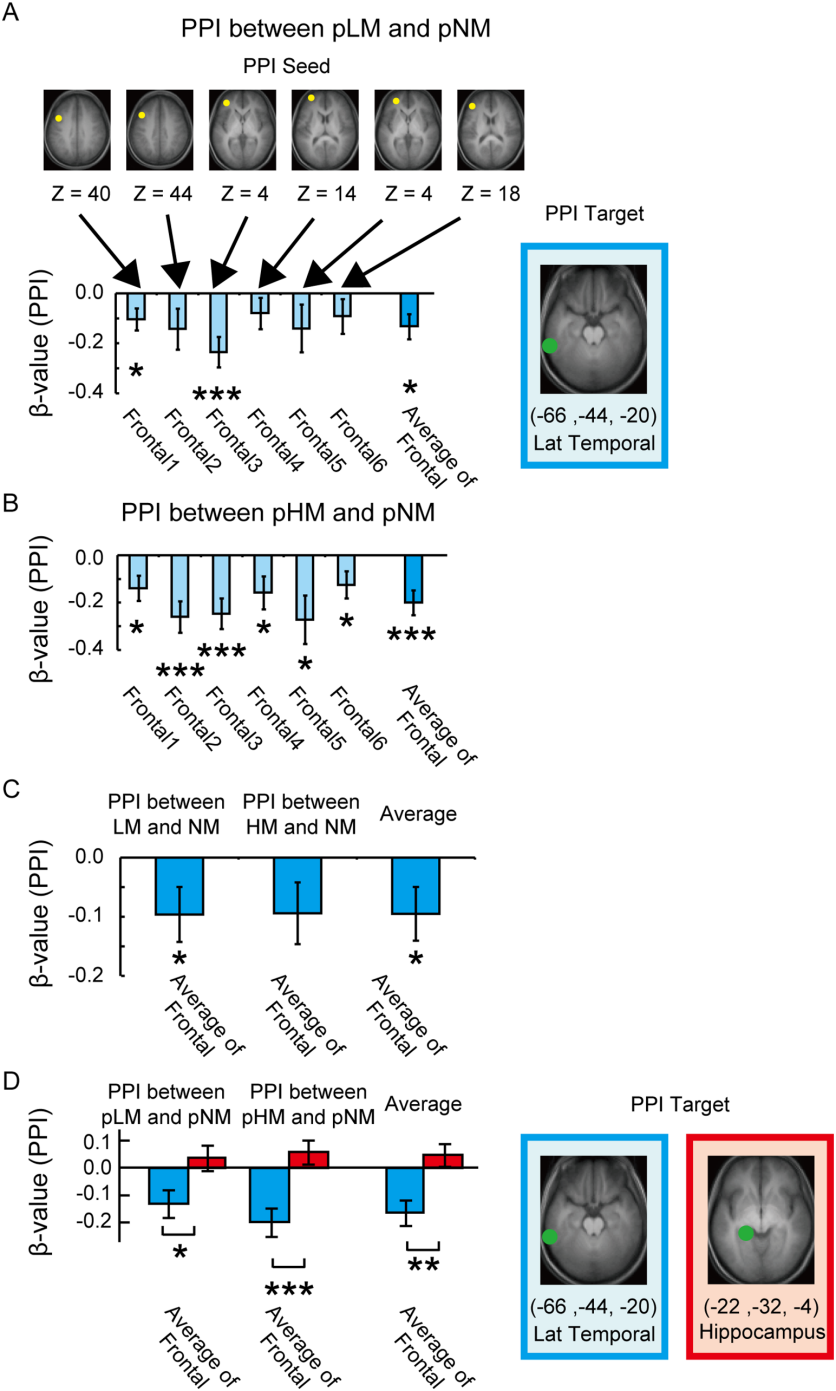
Inhibitory fronto-temporal interaction revealed by a PPI analysis. A) PPIs between pLM and pNM from the lateral prefrontal to the lateral temporal regions. B) PPIs between pHM and pNM from the same lateral prefrontal to the same lateral temporal regions. C) PPIs between LM/HM and NM from the same lateral prefrontal to the same lateral temporal regions. D) PPIs between pLM/pHM and pNM from the same lateral prefrontal to the lateral temporal/hippocampal regions. *: p<.05, **: p<.01, ***: p<.001.

To investigate functional interaction among brain regions during the post-trial fixation periods, we next conducted a psychophysiological interaction (PPI) [45], [46] analysis. Six PPIs were calculated between two psychological conditions, pLM and pNM, from each of the six ROIs in the left lateral prefrontal cortex to the one ROI in the left lateral temporal cortex determined by the contrast of pLM vs. pNM (Fig. 4A). As shown in Fig. 4A, overall tendency of negative PPIs from the lateral frontal regions to the lateral temporal region was observed, suggesting decreased fronto-temporal interaction during the post-fixation periods after the memory trials. To summarize the six frontal ROIs, a PPI from the averaged frontal regions to the temporal region was significantly negative [t (30) = −2.6, P<.05]. In order to test the reproducibility of the negative PPIs, PPIs were also calculated between pHM and pNM in the same ROIs based on the contrast of pLM vs. pNM (Fig. 4B). We found consistent tendency of negative PPIs, and the PPI from the averaged frontal regions to the temporal region was significantly negative [t (30) = −3.8, P<.001]. The PPI from the averaged frontal regions to the temporal region was also significantly negative when pLM and pHM were averaged [t (30) = −3.5, P<.005]. Further, to test whether the functional interaction after the memory trials was also present during the memory trials, the PPIs were calculated between LM/HM and NM in the same ROIs based on the contrast of pLM vs. pNM. The negative PPI from the averaged frontal regions to the temporal region was significant between LM and NM [t (30) = −2.1, P<.05], close to significance between HM and NM [t (30) = −1.8, P = .08], and significant when HM and LM were averaged [t (30) = −2.1, P<.05].

As a reference, we also examined functional interaction between the left lateral prefrontal cortex and the left hippocampus (Fig. 4D). A PPI was calculated between pLM and pNM, from the average of the six ROIs in the lateral prefrontal cortex to the one ROI in the hippocampus determined by the contrast of pLM vs. pNM. The PPI was close to 0. When compared with the PPI to the lateral temporal cortex, the difference was significant [t (30) = 2.1, P<.05]. In order to test the across-data reproducibility, PPIs were also calculated between pHM and pNM based on the same ROIs of the contrasts of pLM vs. pNM. The difference between the two PPIs was also significant [t (30) = 4.2, P<.001]. When pLM and pHM are averaged, the PPI difference was also significant [t (30) = −3.3, P<.005]. These results suggest that the lateral prefrontal cortex interacted specifically with the lateral temporal cortex during post-trial fixation periods.

**Figure 5:**
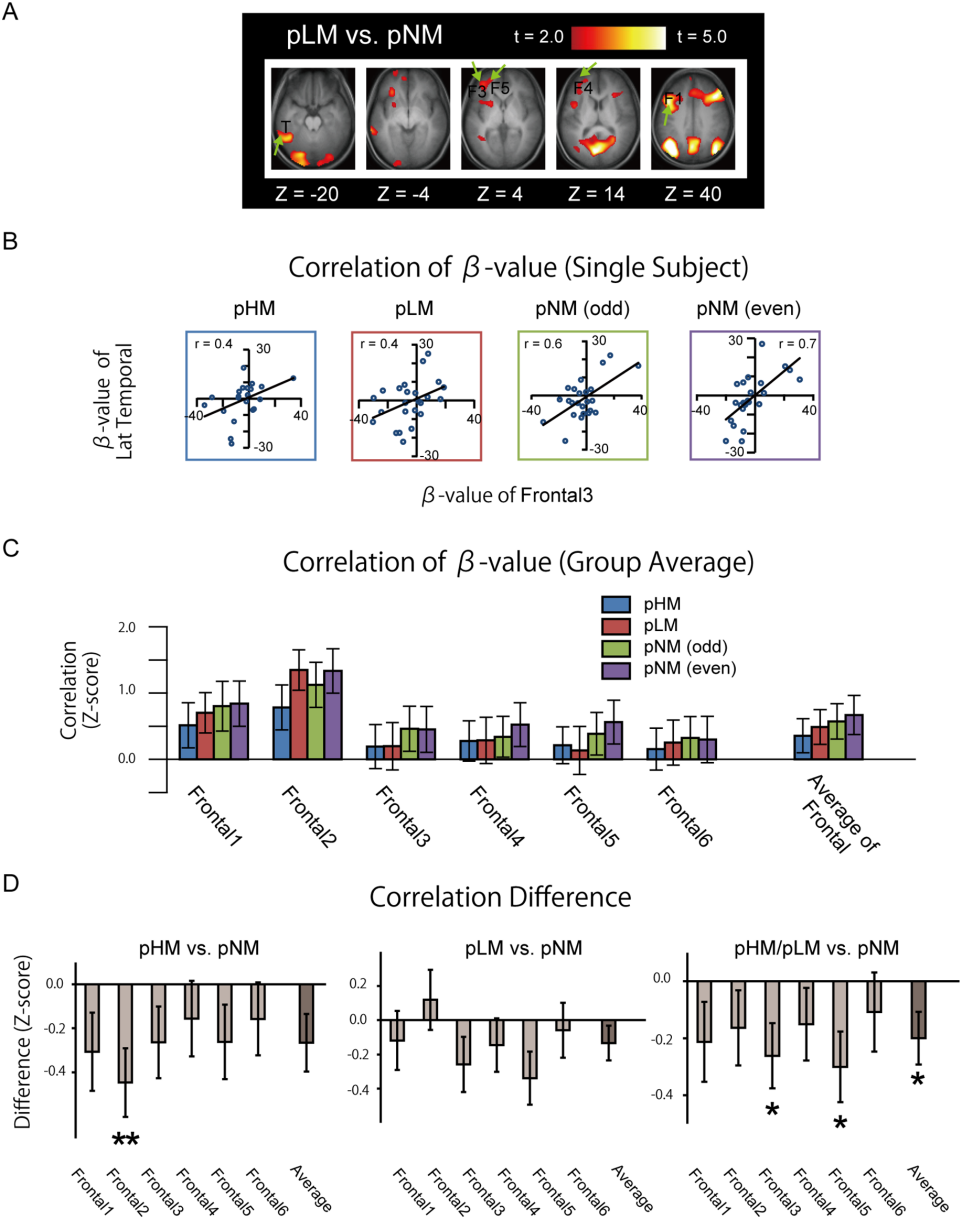
A functional connectivity analysis on a trial by trial basis. A) Activation maps for pLM vs. pNM generated from parameter estimates for individual events. The formats were similar to Fig. 3. B) An example of trial-based functional connectivity between frontal and temporal ROIs during pHM, pLM and pNM (odd and even) in one subject. C) Group average functional connectivity in each of the frontal and the temporal ROIs. D) The connectivity difference between pHM and pNM (left) and between pLM and pNM (middle) and between pHM/pLM and pNM (right). The formats were similar to Fig. 4. *: p<.05, **: p<.01.

The fronto-temporal interaction during post-trial fixation periods was analyzed further using functional connectivity on a trial-by-trial basis [54]. To confirm that the parameter estimates for individual single events were properly calculated, the activation map was re-generated by averaging the parameter estimates for individual trials. Figure 5A demonstrates that, although the overall activation pattern appears relatively weaker, the frontal and temporal activations during pLM vs. pNM that were originally detected by the standard analysis presented in Figure 3 were successfully reproduced using the parameter estimates for individual single events. Next, the parameter estimates for individual single events were plotted against the frontal and temporal ROIs in one particular period. One example is shown in Fig. 5B, where the parameter estimates during pHM/pLM/pNM were plotted against the frontal (Frontal 3, see Table 1) and the lateral temporal ROIs in one subject. To match the number of plots in each period, pNM was divided into two halves (odd and even). The fronto-temporal interaction was investigated thoroughly, calculating the group average for every combination of the six frontal ROIs and the lateral temporal ROI during pHM, pLM and pNM (odd and even) (Fig. 5C). The difference in functional connectivity was calculated between pHM/pLM and pNM (averaged for odd and even) (Fig. 5D), to compare the results with those by the PPI analysis presented in Fig. 4. The difference between pHM/pLM and pNM when six frontal ROIs were averaged was significantly negative [t (30) = 2.2, P<.05]. Importantly, although the difference in functional connectivity was negative, the functional connectivity during each period was positive. That is, the positive fronto-temporal functional connectivity during pNM decreased during pHM/pLM.

**Figure 6:**
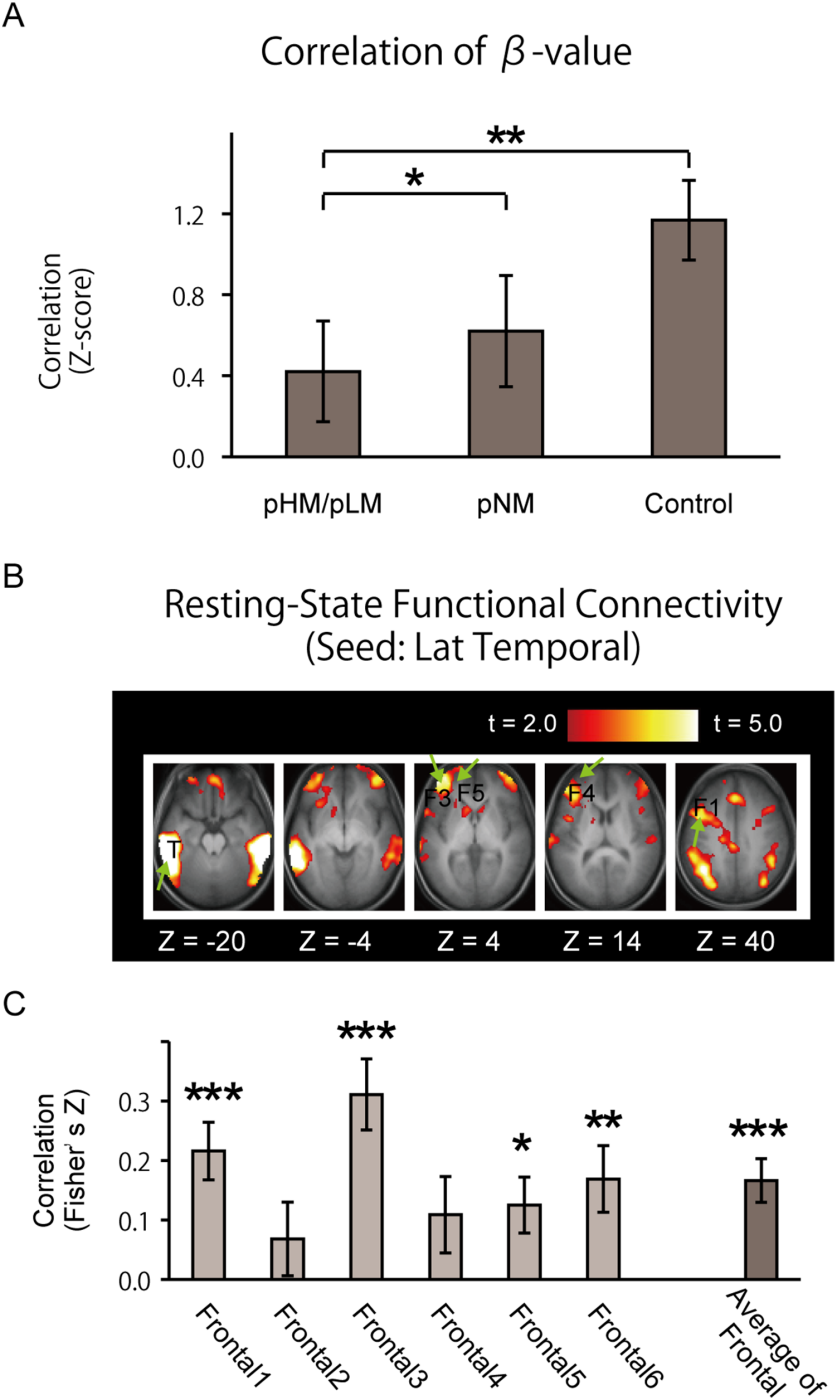
A) A trial-based functional connectivity during pHM/pLM, pNM and control fixation periods. B) A map of resting-state functional connectivity, with the seed placed in the lateral temporal region. The formats were similar to Fig. 3. C) The magnitude of the resting-state functional connectivity between each frontal and temporal ROIs. The formats were similar to Fig. 4. *: p<.05, **: p<.01, ***: p<.001.

Although the memory-related processes during pNM are expected to be minimal (Fig. 2C), we further examined control periods in two runs without any recency judgment trials, where blocks of odd/even judgment trials (NM) and fixation blocks were alternated (see Materials and Methods). Twenty-four events were individually coded in the fixation control periods, and parameter estimates were calculated using similar procedures. Figure 6A demonstrates that the fronto-temporal functional connectivity during the control period was positive, and the difference was significantly negative between pHM/pLM and pNM [t (30) = 2.2, P<.05] and between pHM/pLM and control periods [t (30) = 2.8, P<.01]. To investigate the positive functional connectivity during the control fixation periods, a resting-state functional connectivity was calculated further, using four runs where subjects fixated on a hair cross (see Materials and Methods). As shown in Figs. 6B and 6C, the resting-state functional connectivity between the frontal and temporal ROIs was significantly positive [t (25) = 4.5, P<.001].

## Discussion

In the present fMRI study, we tested whether the fronto-temporal interaction that is known to occur during memory retrieval occurred even during the fixation periods where the memory trials were completed. The contrast of the post-trial fixation periods (pLM vs. pNM) in our modified recency judgment task revealed the heightened brain activity in several regions including the lateral prefrontal cortex, the lateral temporal cortex and the hippocampus. A PPI analysis revealed significant negative functional interaction from the lateral prefrontal to the lateral temporal cortex, but not to the hippocampus. The PPI was also significant during the memory retrieval trials. A trial-based functional connectivity analysis revealed positive fronto-temporal interaction that decreased during pLM relative to pNM. Moreover, a resting-state functional connectivity analysis revealed a positive fronto-temporal functional connectivity during the resting state. These results suggest that at least one part of the top-down prefrontal processing that occurred during memory retrieval was extended into the subsequent fixation periods, and raise the possibility that the post-task fixation periods may not be regarded as an ideal low-level control.

The present study revealed that the fronto-temporal functional connectivity was positive during control fixation periods, and the functional connectivity decreased during post-retrieval fixation periods. The decrease of the positive functional connectivity implies two straightforward interpretations: One is that the prefrontal control over the temporal cortex yields positive functional connectivity, and the prefrontal control is weakened during post-retrieval fixation periods, relative to the control fixation periods. The other is that the positive functional connectivity during the control fixation periods simply reflects spontaneous between-regional correlation that is irrelevant of prefrontal functioning, and the decrease of the functional connectivity reflects enhanced inhibitory prefrontal control over the temporal cortex. The present results from the resting-state functional connectivity, which is known to reflect primarily the anatomical connections [58]–[59], support the latter possibility. One important feature of the present observation is that the brain activity in the lateral temporal region was enhanced during pLM, but the lateral temporal activity was suppressed at the same time through enhanced inhibitory prefrontal control over the temporal cortex during pLM. One straightforward explanation for this counterintuitive pattern of brain activity and PPI would be that memory representation in the lateral temporal cortex was activated by memory retrieval processes within the non-frontal brain regions, and the brain activity surpassed the prefrontal inhibitory interaction.

The posterior lateral temporal cortex has been implicated in specific semantic knowledge [60]–[71]. The post-task questionnaire in the present study revealed that the subjects thought about previous memory trials during fixation after recency judgments, i.e., the stories made from the words in the study list. The stories were made of specific semantic relations between the study words, but semantic relations in the stories had to be selected from other irrelevant semantic relations. It has been proposed that the lateral prefrontal cortex is engaged during post-retrieval processing of semantic information through monitoring and decision making processes [72]–[75]. It has also been proposed that the lateral prefrontal cortex, especially the inferior prefrontal cortex, subserves selection of semantic information among competing alternatives [1], [2], [8]. Thus, the decrease of functional connectivity from the lateral prefrontal cortex to the lateral temporal cortex can be interpreted as post-retrieval processing of selecting relevant semantic representations by inhibiting irrelevant representations through monitoring and decision making processes.

Mental activity during fixation is most often associated with default mode network [76]–[78]. The hippocampus and the lateral temporal cortex reported in the present study appear to belong to the default mode network, and the lateral prefrontal cortex also appears to belong to the fronto-parietal control network [79], [80]. The decrease of fronto-temporal functional connectivity during post-retrieval processing revealed in the present study is consistent with decreased coupling between the fronto-parietal control network and the default mode network during control processing in the pLM [81], [82]. Moreover, the connectivity decrease was greater in the pHM when the control demand was heightened relative to pLM. Although full understanding of cognitive processes recruited during fixation periods needs further research, the present study suggests the fronto-temporal interaction that was extended temporally into the subsequent fixation periods.
